# Microalgae for the Food Industry: From Biomass Production to the Development of Functional Foods

**DOI:** 10.3390/foods11050765

**Published:** 2022-03-07

**Authors:** Tomás Lafarga, Gabriel Acién

**Affiliations:** 1 Department of Chemical Engineering, University of Almeria, 04120 Almería, Spain; facien@ual.es; 2 Functional Unit Desalination and Photosynthesis, CIESOL Solar Energy Research Centre, UAL-CIEMAT, 04120 Almería, Spain

The human population is expected to reach 9.5 billion people by 2050. Feeding this expanded population presents the enormous challenge of doubling food production. This is an enormous challenge, not only because of technological limitations, but also because this production increase must be carried out whilst increasing the sustainability of current processes and protecting biodiversity. Food production is a major driver of biodiversity loss, land usage and the depletion of water resources. Microalgae are shown to be one of the pillars of the future sustainable production of food. At the beginning of the 21st century, microalgal biomass was suggested as a possible source of biofuels; this, together with the high oil prices we are currently experiencing, triggered large investments in microalgal biotechnology. Unfortunately, the lipid yields of real processes are far behind the predictions of theoretical values, but the huge investments made during the last two decades ignited the development of microalgae-based processes with different goals. These include wastewater treatment, CO_2_ capture, and the production of aquafeeds and valuable agricultural products such as biostimulants. However, some challenges still need to be overcome. Significant investments and high operational costs limit the utilisation of microalgal biomass to niche markets, where the high cost of the product compensates for high production costs. These include food supplements and functional foods, which are well accepted by consumers and have a market share that is increasing every year. 

Microalgae are a source of valuable foods and ingredients including proteins, lipids, and bioactive compounds with different bioactivities. Protein is crucial for human health. The use of microalgae as a protein source has been highlighted for different reasons. The production of microalgal protein using photobioreactors is generally more efficient than cultivating plants or raising animals [1]. Not only because of their higher photosynthetic efficiency or a better use of nutrients (nitrogen, phosphorus, etc.), but also because of the potential to control the process and produce food during the whole year. Moreover, photobioreactors can be installed in non-arable land and even in urban areas. Microalgae can also be produced using non-drinkable water (e.g., wastewater), although this strategy is only valid for non-food applications, such as the simultaneous treatment of wastewater and the production of agricultural products. In addition, marine microalgae can be produced using seawater; these strains are rich in polyunsaturated fatty acids as well as other proteins. Other advantages of microalgal proteins are their balanced amino acid compositions and high digestibility; moreover, microalgal proteins are safe as some strains, including *Arthrospira* and *Nostoc,* have been consumed as food for centuries [1]. Further studies are needed to assess the safety and nutritional value of other microalgal strains, as this information would facilitate the commercialisation of microalgal biomass, currently restricting the commercialisation of microalgae-containing foods. Only a limited number of strains have achieved commercial success. These are *Arthrospira platensis,* commercially known as Spirulina, and *Chlorella vulgaris*, which are used as food; *Haematococcus pluvialis* and *Dunaliella salina*, which are used as a source of valuable carotenoids; and marine strains of the genera, *Nannochloropsis, Tetraselmis* and *Isochrysis,* which are used as animal feeds. *Tetraselmis chuii* is now commercialised as a human food in the EU. The microalga *H. pluvialis* is especially interesting, as it produces and accumulates astaxanthin, which was recently suggested to be one of the three main food ingredients ripe for new research and a niche food ingredient with huge potential. Different foods and supplements containing astaxanthin derived from *H. pluvialis* are currently commercially available as well as feed ingredients that increase the characteristic colour of salmon. Natural astaxanthin is a functional ingredient with proven health benefits, most of them attributed to its exceptionally high antioxidant capacity, which surpasses that of other known natural compounds [2]. 

Microalgae are not only rich in proteins and carotenoids but also other valuable bioactive ingredients such as vitamins. Vitamin E, for example, comprises a group of eight lipid-soluble compounds, including tocopherols and tocotrienols, that are synthesised by photosynthetic organisms. The compound α-tocomonoenol was mainly identified in terrestrial plants; however, a recent work detected α-tocomonoenols in microalgae, with 11′-α-tocomonoenol being the predominant isomer in *Chlorella sorokiniana*, *Nannochloropsis limnetica*, and *Tetraselmis suecica* [3]. Other valuable microalgae-derived bioactive compounds include polyunsaturated fatty acids, phycobiliproteins, and polyphenolic compounds, and their use in the food industry is gaining increased interest. The bioactive compounds produced by microalgae are generally accumulated inside their cells. Therefore, a cell wall disruption step is generally needed to either recover and purify this compound or promote bioavailability upon consumption. As of now, many different strategies have been assessed, with high-pressure homogenisation, sonication, and enzymatic hydrolysis among the most widely used. Other strategies, such as pulsed electric fields or bead milling, have also been assessed as steps to disrupt microalgal cell walls. These methods can be used alone or combined; generally, a combination of a physical and a chemical or biological process leads to a synergetic effect. Indeed, in a recent study, lipids rich in omega-3 fatty acids were extracted from the microalga *Nannochloropsis gaditana* using a combination of ultrasound and enzymatic hydrolysis, with the authors of the study reporting a synergetic effect between both strategies. The combination of ultrasound (37 kHz, 140 W) with enzymatic hydrolysis using the enzyme Viscozyme^®^ (55 °C, 6 h) led to an oil yield of 29%, higher than that obtained when using ultrasound or enzymatic hydrolysis alone [4]. 

Microalgal biomass and microalgae-derived ingredients are being used for the production of a wide variety of foods. The most promising food matrices to use as delivery vehicles of microalgae, in terms of consumer acceptance, are baked products, pasta, and sauces or seasonings. Different works demonstrated the potential of microalgae to enrich the nutritional value of baked products. For example, the incorporation of *Tetraselmis chuii*, *Nannochloropsis gaditana*, and *Chlorella vulgaris* into gluten-free bread increased their protein, lipid, and mineral (Ca, Mg, Cu, Zn, K, P) contents [5]. In that study, the organoleptic attributes of the product were negatively affected after introducing microalgal biomass; however, the authors were able to obtain a highly nutritious product with improved technological and organoleptic attributes by including in the process a pre-treatment of biomass using ethanol. More studies assessing the effect of microalgal biomass on the physical, chemical, organoleptic, and bioactive properties of foods are necessary to promote the utilisation of microalgae and private investments to trigger the consumption of this valuable resource. 

Overall, microalgal biomass is gaining an increasing interest as a food ingredient. However, the number of products that are launched into the market and achieve commercial success is very limited. Further studies, as well as marketing campaigns to increase consumers’ awareness of the positive environmental and health outcomes of producing and consuming microalgae, are vital. Microalgae are rich in sustainable and health-promoting compounds, and their utilisation in food will certainly increase in coming years.
